# Proving the robustness of a PEDOT:PSS-based thermistor *via* functionalized graphene oxide–poly(vinylidene fluoride) composite encapsulation for food logistics†

**DOI:** 10.1039/d0ra00554a

**Published:** 2020-03-26

**Authors:** Bijendra Bishow Maskey, Kiran Shrestha, Junfeng Sun, Hyejin Park, Jinhwa Park, Sajjan Parajuli, Sagar Shrestha, Younsu Jung, Subramaniyan Ramasundaram, Gyan Raj Koirala, Gyoujin Cho

**Affiliations:** Sungkyunkwan University, Department of Biophysics 2066, Sebu-ro, Jangan-gu Suwon-si Gyeong gi-do Republic of Korea gcho1004@skku.edu grkoirala@skku.edu; Korea Institute of Science and Technology, Center for Water Resource Cycle Research (CWRC) 5, Hwarang-ro, Seongbuk-gu Seoul 02792 Korea

## Abstract

The instability of poly(3,4-ethylenedioxythiophene):polystyrene sulfonate (PEDOT:PSS) under a humid condition is the major limitation in the practical development of a flexible thermistor. Here, we introduced a functionalized graphene oxide–polyvinylidene fluoride (FGO–PVDF) composite as an encapsulation layer to prove the reliability of PEDOT:PSS thermistors under high-humidity conditions. The FGO–PVDF-encapsulated thermistor exhibited good linearity, a resolution of 1272.57 Ω per °C, a temperature coefficient of resistance equal to −3.95 × 10^−3^ per °C, stable performance, and an acceptable response time (∼40 s per °C) calibrated in the temperature range between −10 °C and 30 °C, resembling the temperature of a cold chain system. For applications in a food cold chain system, this thermistor was integrated into a roll-to-roll (R2R) gravure-printed NFC antenna, a microcontroller-embedded Si-chip transponder, and a printed battery to work as a smart label to wirelessly monitor the time–temperature history (TTH) of a food package. A proof-of-concept study was demonstrated by attaching an NFC-enabled hybrid TTH logger, a smart label, in a chicken package.

## Abstract 2

We introduced a FGO–PVDF composite as an encapsulation layer to prove the reliability of PEDOT:PSS thermistors under high-humidity conditions to realize an NFC-enabled smart label for monitoring time-temperature history of a food item along the cold chain.

## Graphical abstract

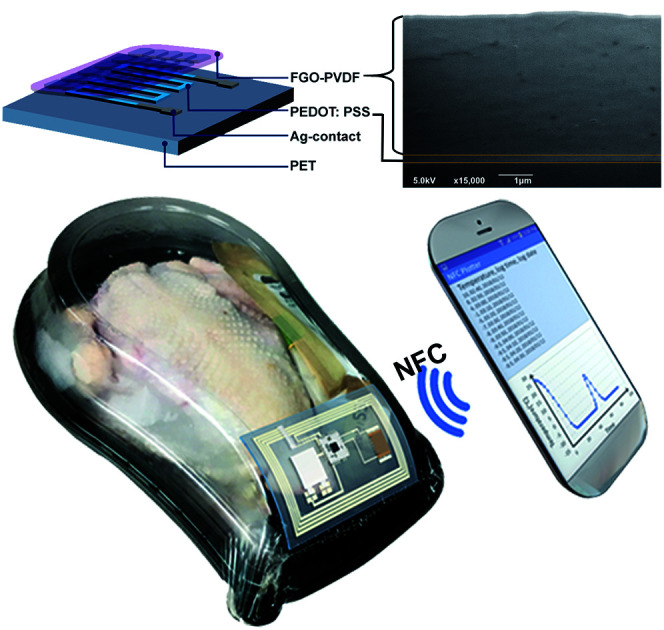

## Introduction

The intrinsic capability of conducting polymers, mainly derived from polyaniline, polypyrrole, polythiophene, and poly(3,4-ethylenedioxythiophene), whose electrical and optical properties can be tailored by controlling the doping state, is pivotal to their widespread applications in sensors, displays, *etc.*^1–3^ Among them, the poly(3,4-ethylenedioxythiophene):polystyrene sulfonate (PEDOT:PSS) electroactive polymer has been attracting attention owing to its low-cost processing, high conductivity (∼10^3^ S cm^−1^), optical transparency, improved wettability, and compatibility with the emerging printing technology.^4^ Consequently, PEDOT:PSS and its derivatives have been extensively studied for realizing flexible and transparent electrodes for organic light-emitting diodes (OLEDs),^5^ supercapacitors,^6^ sensors,^7^*etc.* In particular, PEDOT:PSS exhibits temperature-dependent resistivity, enabling the possibility of realizing polymer-based semiconducting thermistors. Indeed, the temperature-dependent resistivity of PEDOT:PSS is governed by Mott's variable range hopping (VRH), where the logarithmic value of resistance is inversely proportional to the activation energy of localized charge carriers.^8–10^ This inverse logarithmic relationship exhibits a negative thermal coefficient (NTC) and is more accurate for the detection of temperature.^11^ As compared to the oxide ceramic-based NTC thermistors requiring high sintering temperatures (1200–1300 °C), polymer thermistors can be cured at low temperatures and are solution processable. Moreover, a unique feature of polymer-based thermistors is their ability to adjust to a high base resistance (from hundreds of kΩ to MΩ), leading to the enhancement in their resolution per °C; this in turn improves the signal-to-noise ratio of the sensor. However, the instability of printed PEDOT:PSS thermistors is one of the challenging issues to be addressed, especially in a high humidity environment where the resistance of the thermistors increases due to the strong hygroscopic nature of PSS.^8,12^ As a result, previously, the PEDOT:PSS thermistors have been characterized under an inert environment and therefore, their practical applications are limited.^12^ Furthermore, despite showing a relatively robust response against humidity, the reported tosylate-doped PEDOT (PEDOT:tos) thermistor shows instability at lower temperatures (<10 °C)^8^ and therefore is not suitable for applications in a cold chain system. Thus, a reliable encapsulation material that can withstand even negative temperatures equivalent to those in a cold chain should be developed to enhance the stability of PEDOT:PSS-based thermistors. In this regard, several efforts, such as substitution with polymeric ionic liquids^8,13^ or use of additives, such as sorbitol^14^ and dimethylsulfoxide (DMSO)^15^ with high boiling points, have been made to stabilize the performance of PEDOT:PSS; however, none of the reported studies have been able to circumvent the effect of humidity.

**Fig. 1:**
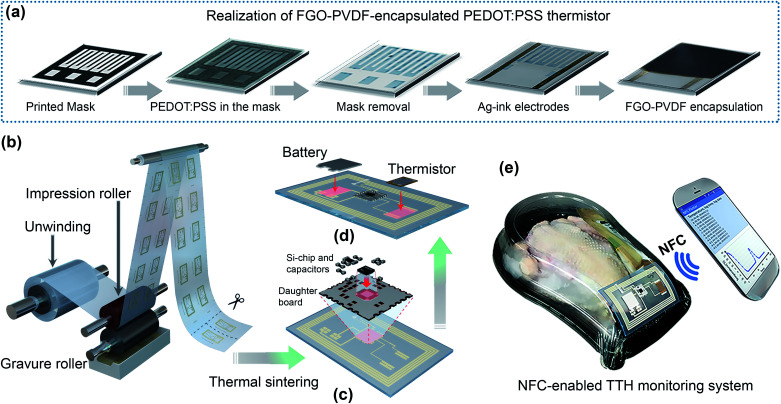
Schematic of realizing a time–temperature history (TTH) monitoring system. (a) Realization of the FGO–PVDF-encapsulated PEDOT:PSS thermistor by a simple printing process. (b) Schematic of the R2R gravure printing machine. Assembly of Si-chip components using a daughter board (c) and a printed battery and thermistor (d) to the printed circuit. (e) Concept of utilizing the NFC-enabled TTH-monitoring system in a chicken package.

Recently, graphene and graphene oxide have been proposed as surface passivation materials for organic as well as silicon (Si) solar cells and as coating materials for metals owing to their impermeability and thermal/chemical stability to reactants.^16–18^ Furthermore, the blending of graphene derivatives with polymer materials would enhance the mechanical strength, integrity, and stability of the encapsulated layer. Polyvinylidene fluoride (PVDF) is a hydrophobic polymer with a greater chemical and thermal stability and an ideal polymeric membrane for application in aqueous solutions; thus, it can be effectively blended with functionalized graphene oxide (FGO) for application in wearable devices.^19^ However, to the best of our knowledge, to date, no study has been reported on the development of FGO–PVDF-encapsulated conducting polymer-based sensors to prevent water permeation. In this study, we demonstrated the utilization of FGO–PVDF as an encapsulation material for a PEDOT:PSS-based thermistor (hereinafter abbreviated as FGO–PEDOT-based thermistor) and successfully applied this thermistor to monitor the temperature range analogous to that of the cold chain. Fig. 1a illustrates a detailed process, starting from mask printing to encapsulation *via* the FGO–PVDF composite, of realizing the FGO–PEDOT thermistor.

Monitoring the time–temperature history (TTH) of the cold chain is crucial as according to the statistics of the Centres for Disease Control and Prevention, most outbreaks of foodborne disease occur as a result of mishandling of food, especially under incongruous time–temperature conditions, in the cold chain.^20^ Therefore, an inexpensive and reliable intelligent packaging system embodying the advantages of both conventional silicon-based electronics for memory and the emerging printed electronics for the low-cost manufacturing of the associated components to realize a flexible hybrid electronic platform is required.^21–23^ In this regard, we incorporated a single-step roll-to-roll (R2R) gravure system for printing antenna and interconnect lines in poly(ethylene terephthalate) (PET) and polyimide (PI) rolls, respectively (Fig. 1b). Each individual antenna was subjected to thermal treatment and then assembled with a Si-chip transponder utilizing a daughter board (Fig. 1c); subsequently, the printed battery and FGO–PEDOT thermistor were assembled (Fig. 1d). The fully assembled tag was attached to a chicken package to monitor the TTH, and the logged data were retrieved using an NFC function of the smartphone and plotted using a custom-developed Android application (Fig. 1e).

## Experimental

### Fabrication of the FGO–PEDOT thermistor

Initially, a mask was printed on a PET film using a commercial monochrome laser printer (Sindoh LP 430 dn). The hydrophilic end of PEDOT:PSS allows it to print the gap between the hydrophobic carbon masks. The printability of PEDOT:PSS was enhanced by cleaning the PET surface with detergent water, washing with ethanol, and then drying with a hot air blower. The commercially available PEDOT:PSS (Clevios PH 1000, OMNISCIENCE) was printed on the mask-patterned PET film using bar coating (RDS 5 bar coater) and subsequently dried in an oven at 125 °C for 15 min. The pattern was then washed with toluene (Sigma Aldrich) to remove the laser-printed carbon mask. Herein, the excess solvent was evaporated using the hot air blower followed by vacuum drying at 125 °C for 10 h. The printed thermistor was washed with *N*,*N*′-dimethylformamide (DMF) (Sigma Aldrich) and dried in a vacuum oven at 125 °C for 2.5 h to enhance its surface morphology for improved encapsulation.

**Fig. 2:**
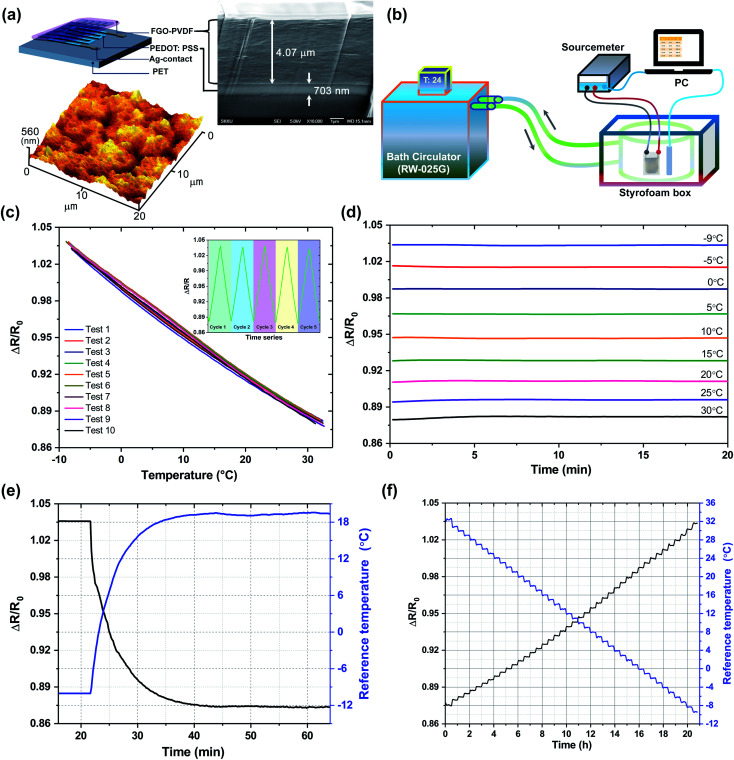
Characterization of the thermistor. (a) Cross-section view of the proposed thermistor comprising the FGO–PVDF-encapsulated PEDOT:PSS layer in a flexible polyethylene terephthalate (PET) substrate. The atomic force microscopy (AFM) image reveals the surface roughness of the encapsulation ink. (b) The custom-designed measurement system to characterize the performance of the printed thermistor. (c) Sensitivity and hysteresis test from −8 °C to 30 °C in five different cycles (inset figure). (d) Stability test at different temperatures measured for 20 min. (e) Response time tested for a period exceeding 1 h. (f) Resolution of the sensor (determined to be at least 1 °C by continuous test measurement for approximately 21 h).

The FGO–PVDF encapsulation composite was prepared by first oxidizing graphite (45 μm, Sigma-Aldrich) by a modified Hummers' method.^24^ The resultant graphite oxide (0.5 g) was stirred with DMF (80 mL) and phenyl isocyanate (Sigma Aldrich) (2.8 g) at room temperature (∼26 °C) under an argon atmosphere.^25,26^ The resulting mixture was worked up using dichloromethane (CH_2_Cl_2_) (Sigma Aldrich) to yield the amide and carbamate ester-functionalized graphite oxide powder. After treatment with phenyl isocyanate, the graphite oxide powder was sonicated with DMF and centrifuged to obtain an FGO dispersion with a solid content of 3 mg mL^−1^. A typical encapsulation composite was formed by dissolving 1.0 g of PVDF (*M*_w_ = 180 000, Sigma-Aldrich) in 20 mL of FGO–DMF dispersion. The mixture was stirred at 75 °C for 12 h and homogenized by sonicating for 10 min. The PEDOT:PSS thermistor was first painted with Ag-ink (Inktech Co. Ltd., Korea) to create a contact terminal, then encapsulated under inert conditions inside a glovebox by drop casting, and finally, subjected to slow heating on a hot plate at 120 °C to allow the excess solvent to evaporate. Thus, the encapsulated FGO–PEDOT thermistor comprised a PET substrate, a PEDOT:PSS thermistor, Ag electrodes, and an FGO–PVDF encapsulation layer (Fig. 2a).

### Silver ink preparation and R2R gravure printing process

Conducting ink based on silver nanoparticles (AgNPs) was formulated using silver gel (PARU, PD001) and diethylene glycol (Daejung, Korea) as a vehicle such that the viscosity was 900 cP and the surface tension was 43 mN m^−1^ (ESI Fig. S1†). Then, a single-step R2R gravure system was used to print a 4-turn NFC antenna and interconnect lines on PET and PI rolls, respectively, under the same printing conditions: a printing speed of 6 m min^−1^, a nip-roll pressure of 6 kgf, and a blade angle of 9°.

### Characterization and performance measurement

The cross-section image was obtained by a field-emission scanning electron microscope (FESEM, JSM-7000F, Jeol Ltd.), and the surface roughness was examined by atomic force microscopy (AFM, AFM5100N, Hitachi High Technologies Co. Ltd.). A customized measurement setup comprising a bath circulator (RW-025G), a source meter, glass vessel *etc.* was utilized for examining the performance of the thermistor (Fig. 2b). The bath circulator was controlled by Lab Tracer (v 2.2), which regulated the temperature through the glass jacket, and the corresponding resistance of the proposed thermistor was simultaneously determined using the Keithley 2400 source meter along with a reference temperature sensor rod. An optical microscope (MM6C-DC310-2, Olympus Co., Japan) was used to measure the print quality of the R2R gravure-printed NFC antenna and interconnect patterns. The thickness of the printed antenna was measured by a surface profiler (NV-220, Nanosystem, Korea).

## Results and discussion

The cost fraction could be substantially reduced in the conventional Si-chip devices by replacing the existing thermistor with a printed thermistor and bridging the Si-chip with an R2R-printed circuit.^23^ Naturally, the PEDOT:PSS ink employed herein to realize the printed thermistor is R2R printable and can offer a fully gravure-printed sensor device in the future. In fact, recent studies have successfully demonstrated the printability of PEDOT:PSS as a transparent electrode material *via* screen^5^ and inkjet^27^ printing. PEDOT:PSS-based thermistors exhibit negative temperature coefficient behaviour because fewer PSS microparticles exist at high temperatures and this reduces the resistance of the thermistors; however, the conducting PEDOT does not have sufficient energy to overcome the PSS boundaries at low temperatures, resulting in an increased resistance.^28^ The increased resistance at negative temperatures is attributed to the formation of ice crystals that restrict the free movement of charge carriers.^29^ Furthermore, at low temperatures, only inter-molecular hopping transport is possible due to the spatial localization of conducting electrons at the Fermi level.^30^ However, temperature-dependent resistance of the printed PEDOT:PSS thermistor is highly sensitive to humidity, which results in extensive hysteresis and inconsistent performance under ambient conditions.^28^ The charge carrier mobility is influenced by humidity as the resistance will increase with an increase in the tunnelling distance in PEDOT chains depending upon the hydrophilic PSS content.^8^ A clear evidence of this increased resistance was observed in our PEDOT:PSS-based thermistor calibrated without any encapsulation (ESI Fig. S2†).1
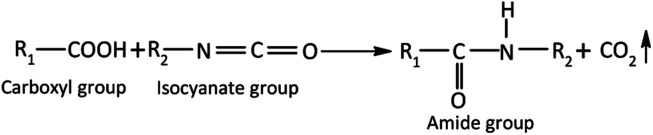
2
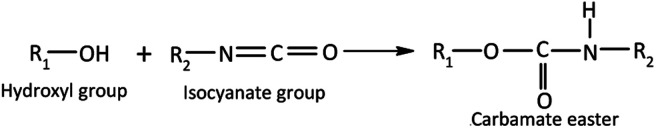


Our approach to resolve the susceptibility of PEDOT:PSS-based thermistors to humidity involves the development of a printable encapsulation layer formulated using FGO in DMF and the PVDF polymer.^25,26,31^ When treated with phenyl isocyanate, the carboxyl and hydroxyl groups of graphite oxide (GrO) are converted into amide and carbamate ester groups (eqn (1) and (2)), respectively, which are incapable of forming hydrogen bonds with water molecules and thereby suppressing the hydrophilicity of GO.^25,26^ Indeed, the successful conversion of GrO to these ester groups *via* treatment with the phenyl isocyanate reagent was confirmed *via* Fourier transform infrared spectroscopy (FTIR) and X-ray photoelectron spectroscopy (XPS). The new peak emerging at 1703 cm^−1^ in the FTIR spectra is associated with C=O of carbamate esters, whereas the loss of a peak at 1732 cm^−1^ indicates the conversion of the carboxyl group into an amide group. Furthermore, the N 1s peak at 400.1 eV associated with CONH was observed by XPS.^26^

Owing to its layered structure and hydrophobicity, FGO can effectively cover and protect the moisture-sensitive electronic device components made up of PEDOT:PSS. Meanwhile, FGO can be well dispersed in polar aprotic solvents, such as DMF, which is also a good solvent for dissolving PVDF, due to the modification of epoxy groups on the surface of graphene oxide through ring opening reactions.^32^ The homogeneous dispersion of FGO along with PVDF, having a lengthy backbone due to its high molecular weight, restricts the sheet-to-sheet contact of FGO, thereby ensuring mechanically robust hydrophobic insulation. Furthermore, the removal of carboxyl and hydroxyl group residues following the annealing process (120 °C for 2 h) under vacuum enhances the cross-linking of the FGO–PVDF encapsulation layers.

Although the amount of the PVDF polymer manipulates the viscosity, dispersion, and mechanical robustness of the encapsulated ink, the amount of FGO should be optimized to ensure the effectiveness of this composite as an encapsulation material. In this regard, we studied three different ratios (2 wt%, 6 wt%, and 10 wt%) of FGO in DMF. A stable cyclic thermo-resistive performance of the FGO–PEDOT thermistor with 6 wt% FGO was obtained as a consequence of the uniform coating of the encapsulation material over the PEDOT:PSS layer and possessing sufficient hydrophobicity (ESI Fig. S3†). An encapsulated thin-layer was formed using a 2 wt% solution, which is not sufficient to isolate the PSS content present in the PEDOT:PSS layer from the external humidity, thereby resulting in instability of the thermistor (ESI Fig. S3b†); on the other hand, the solution with 10 wt% FGO resulted in non-uniform encapsulation due to increased viscosity (ESI Fig. S3a†). Furthermore, despite showing a stable performance, the encapsulation layer would delaminate from the substrate following the annealing process due to its increased thickness. Therefore, further experiments were carried out using the composite solution comprising 6 wt% FGO to characterize the proposed thermistor.

The cross-sectional schematic with the FESEM image of the FGO–PEDOT thermistor realized using a flexible PET substrate is shown in Fig. 2a. The thickness of the PEDOT:PSS layer is 703 nm, whereas that of the drop-casted encapsulation layer is 4.07 μm. Furthermore, it was found that the encapsulation layer was perfectly adhered to the PEDOT surface as no significant defects were noticed between the layers. The average surface roughness values of the encapsulation layer measured at two different positions having an identical surface area of 20 × 20 μm^2^ were found to be 50.76 nm (region 1) and 57.25 nm (region 2), respectively (Fig. 2a, ESI Fig. S4†). The similar surface roughness obtained from two different measurement areas confirms the uniform coating of the encapsulation layer.

Measurement results showed that the resistance of the PEDOT:PSS-based thermistor ranged from 250 to 350 kΩ. Even though it was reported to have a quadratic relationship between the temperature and PEDOT:PSS based thermistor,^33^ we observed a linear temperature–resistance relationship in our study which might be due to the different temperature range (ref. 33: 30 to 170 °C; our work: −10 to 30 °C) of study. As revealed in Fig. 2c, the resulting FGO–PEDOT thermistor showed no hysteresis, whereas a continuous increment in the base resistance values was observed for the thermistor without encapsulation (ESI Fig. S2†). Instead, the FGO–PEDOT thermistor exhibited excellent periodic consistency in temperature-dependent resistance in the temperature range of interest. The average linear resolution, a metric for examining the signal-to-noise ratio, of the thermistor (Δ*R*/Δ*T*) was found to be 1272.57 Ω per °C, which was 50 times that of the reported hydrophobic silver nanoparticle-based thermistor exhibiting a positive thermal coefficient.^23^ Furthermore, the temperature coefficient of resistance (calculated at *T* = 25 °C) of the proposed FGO–PEDOT thermistors was found to be −3.95 × 10^−3^ per °C. This linearity of the thermistor during cyclic measurements ensures that the proposed FGO–PVDF encapsulation layer is highly efficient to circumvent the effect of humidity because there will be periodic formation of ice–liquid–vapor states of water molecules on the inner surface of the glass jacket as a result of temperature variation. We evaluated the stability of the thermistor (Fig. 2d) by monitoring the variation of resistance at −9, −5, 0, 5, 10, 15, 20, 25, and 30 °C for 20 min using a measurement setup. The stability of the passivated thermistor was acceptable and originated from the hydrophobicity of the FGO–PVDF layer at the expense of the response time (approximately 20 min to transit from −10 °C to 18 °C equivalent to ∼40 s per °C, Fig. 2e); this is believed due to the poor thermal conductivity of the encapsulation layer. However, the attained response time of 20 min for an overall temperature range of interest is comparable to the temperature response of the commercially available temperature sensor (TMP 112, Texas Instruments) used as a reference. Furthermore, we believe that the response time of the proposed thermistor is sufficient to monitor the TTH in food packaging since the chances of an abrupt change in temperature in the cold chain are minimum. Therefore, the FGO–PEDOT thermistor can effectively monitor the temperature that varies at a slow rate and for a long period (Fig. 2f). Furthermore, a resolution of 1 °C between the range from −10 to 30 °C (Fig. 2f) is sufficient to deploy with food packaging. Hence, the fabricated FGO–PEDOT thermistor was well optimized for implementation in food packaging as a cost-effective solution for mass food production.

**Fig. 3:**
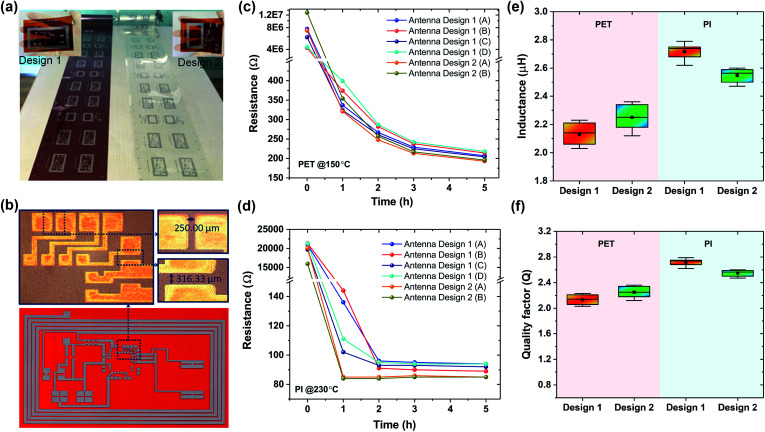
Images and electrical characteristics of the printed NFC antenna. (a) R2R-printed antenna and circuit patterns on PET (right) and PI (left) substrates along a 30 m web. Inset images show the antenna design 1 and 2 printed on a PI roll. (b) Printed antenna on a PI roll and microscopic images of the interconnect lines to which the daughter board is attached. (c and d) Decreasing values of resistance of the antenna designs on PET and PI substrates following constant heat treatment at 150 °C and 230 °C with respect to time, respectively. (e and f) Variation in the inductance and *Q* factor of the printed antenna designs (1 & 2) on PET and PI substrates, respectively.

Silver nanoparticle-based conducting ink was utilized to print six different antenna patterns (four from design 1, labelled from A to D, and two from design 2, labelled from A to B, refer to the ESI Table S1† for details) on both substrates along a 30 m web, as shown in Fig. 3a. The thickness of the printed antenna for the PET and PI rolls varied between 530 nm and 600 nm (ESI Fig. S5†). To bridge the Si-chip with the R2R-printed circuit, the physical dimensions of the contact electrodes were maintained at approximately 500 × 300 μm^2^ with a pattern resolution of 250 μm (Fig. 3b). As shown in Fig. 3c and d, the resistance of the antennas printed on both the PET and the PI rolls was unsuitably high (in the kΩ to MΩ range) and hence limited their practical application. After additional curing as a function of time, the resistance values were found to gradually decrease with the increasing curing temperature. With PI, the resistance became saturated after 2 h of curing at 230 °C, whereas the resistance remained relatively high for the PET substrate even after curing for 5 h at 150 °C. The inductance of the R2R gravure-printed antenna on the PET and PI rolls was ∼2.2 and ∼2.6 μH, respectively (Fig. 3e). The attained *Q* values as a function of the measured resistance (*R*) and inductance (*L*) presented by *Q* = *ωL*/*R* are plotted in Fig. 3f, demonstrating higher *Q* for the designs printed on the PI substrate mainly due to the low resistance of the printed antenna following high thermal curing. The highest *Q* of 2.9 was obtained for the antenna pattern (Antenna Design 1(C)) on PI cured at 230 °C for 2 h, and the induced power could effectively couple with the NFC function of the smartphone.

**Fig. 4:**
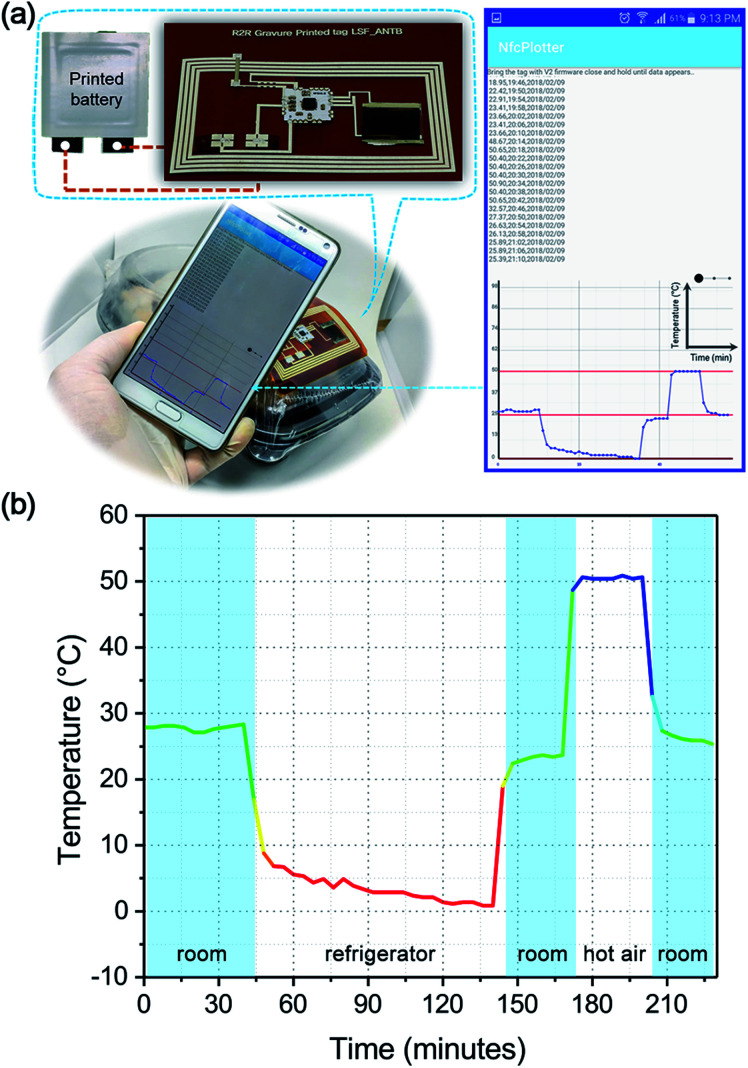
Real-time testing and validation of the TTH monitoring system. (a) The smartphone displays the temperature history log extracted from the NFC thermistor attached to the chicken package. (b) Magnified view of the logged temperature data under different scenarios. Refer to the ESI Video† for the detailed information of the measurement conditions.

The Si-chip was bridged with the R2R gravure-printed circuit using an epoxy-based conducting paste instead of solder such that the Si-chip bonding could be simultaneously carried out after printing in the future. However, at this stage, we demonstrated a way for lowering the manufacturing cost by employing a printed antenna, a printed interconnection circuitry, a printed thermistor, and a printed battery for fabricating the Si-chip-based hybrid NFC-thermistor tag. The bridging process was started by first bridging the Si-chip (RF430FRL152H, Texas Instruments) on a daughter board and consequently bridging the FGO–PEDOT thermistor and printed battery (1.6 V with 30 mA h, provided by Changzhou Institute of Printed Electronics Industry, China). We assembled the Si-chip on the daughter board and stacked it on the printed circuit pattern to maintain the pattern resolution. Indeed, the utilization of a daughter board eliminates the failure rate associated with bridging the Si-chip directly to the printed circuit. The tuning capacitor (10 pF) was mounted on the daughter board such that the antenna resonated at 13.56 MHz when coupled with the smartphone (ESI Fig. S6†). To demonstrate the concept of an intelligent packaging system, the assembled NFC tag was attached to a chicken package bought from the commercial market. The data obtained from the thermistor was accessed by a smartphone *via* an inbuilt NFC function and retrieved from a custom-developed Android-based application (Fig. 4). Extreme temperature conditions along the logistic chain were examined using refrigerator, ambient, and hot air to prove the robustness of the proposed thermistor for the intelligent packaging system (Fig. 4b, ESI Video†).

## Conclusions

Herein, we demonstrated an FGO–PVDF-encapsulated PEDOT:PSS-based thermistor for application in an NFC-enabled intelligent packaging system along the food logistic chain. The experimental results showed that the proposed FGO–PEDOT thermistor was robust in monitoring the TTH along the logistic chain system. The thermistor thus realized was incorporated with the R2R gravure-printed NFC antenna, a Si-chip transponder, and a printed battery to wirelessly monitor the TTH history using the NFC function of the smartphone along with an Android app for data logging and post-processing. Note that the major contributors of this smart label are the Si-chip and the printed battery employed to obtain and post-process the data obtained from the thermistor. It will be too early to specifically estimate the cost per label before optimizing the respective inks compatible with R2R processing for mass production; however, we believe that the overall cost will be less than $1.0 per label (estimated cost of $0.70 per label excluding the labour and operating cost, ESI, Table S2†). Future studies should be focused on the development of fully R2R-printed food labels offering Si-chip bonding in line with all-printed units of NFC antenna, the FGO–PEDOT-based thermistor, and a battery. In this regard, the proposed intelligent packaging system would form the basis for future smart logistics as a part of Forth Industrial Revolution.

## Conflicts of interest

There are no conflicts to declare.

## Supplementary Material

RA-010-D0RA00554A-s001

RA-010-D0RA00554A-s002
