# Supplementary material for: Proving the robustness of a PEDOT:PSS-based thermistor via functionalized graphene oxide–poly(vinylidene fluoride) composite encapsulation for food logistics
Source: RSC Adv. 2020 Mar 26;10(21):12407–14. doi: 10.1039/d0ra00554a (PMC9050635; doi:10.1039/d0ra00554a)
Supplement: RA-010-D0RA00554A-s001 [file RA-010-D0RA00554A-s001.pdf]

Electronic Supplementary Material (ESI) for RSC Advances.
This journal is © The Royal Society of Chemistry 2020

Supplementary Information

Proving the robustness of PEDOT:PSS-based thermistor *via* functionalized graphene oxide-poly(vinylidene fluoride) composite encapsulation for food logistics†

Bijendra Bishow Maskey,[a] Kiran Shrestha,[a] Junfeng Sun,[a] Hyejin Park,[a] Jinhwa Park,[a] Sajjan Parajuli,[a] Sagar Shrestha,[a] Younsu Jung,[a] Subramaniyan Ramasundaram,[b] Gyan Raj Koirala,*[a] and Gyoujin Cho,*[a]

[a] *Sungkyunkwan University, Department of Biophysics,2066, Sebu-ro, Jangan-gu, Suwon-si, Gyeong gi-do, Republic of Korea*
[b] *Korea Institute of Science and Technology, Center for Water Resource Cycle Research (CWRC), 5, Hwarang-ro, Seongbuk-gu, Seoul 02792, Korea.*
**Correspondence: G. Cho (gcho1004@skku.edu), G. R. Koirala (grkoirala@skku.edu)*

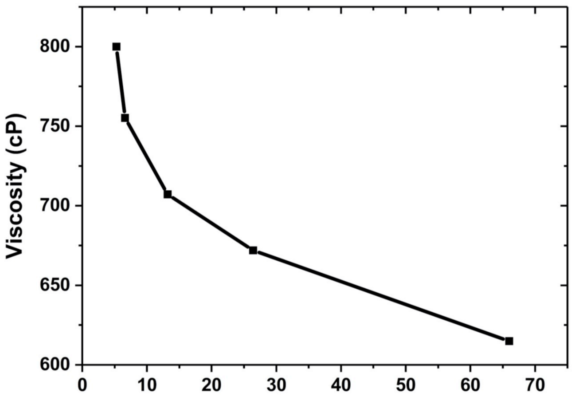

Fig. S1 Viscosity as a function of shear rate for the conducting silver-nanoparticle ink prepared for R2R printing of NFC antenna and circuit pattern.

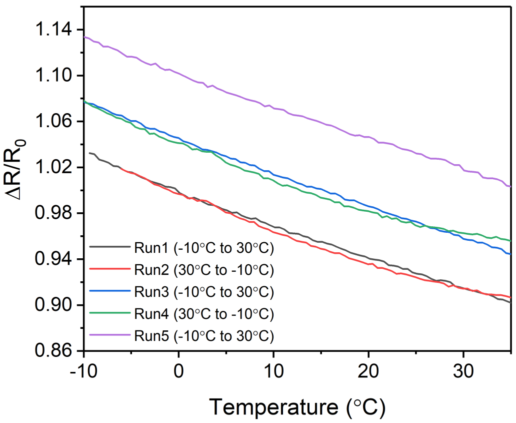

Fig. S2 Stability issues of PEDOT:PSS thermistor for a cyclic measurement between -10°C and 30°C calibrated without any encapsulation..

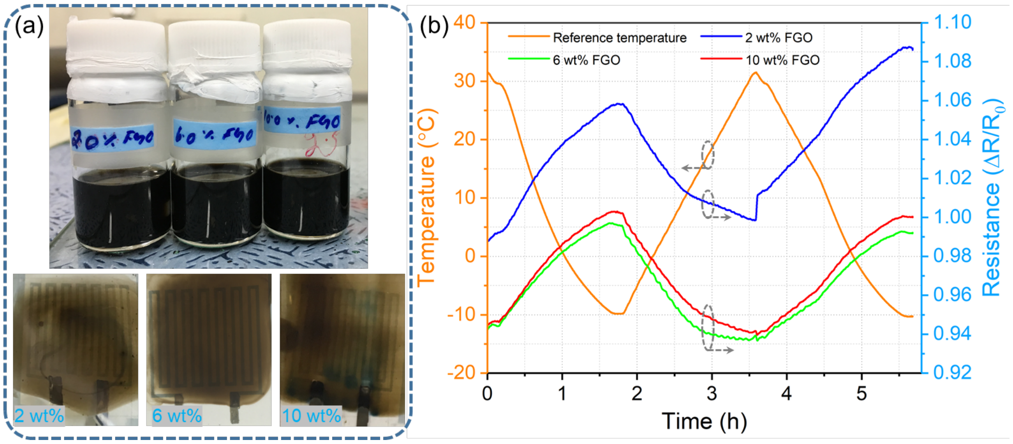

Fig. S3 Optimization of the composite ink for uniform encapsulation. (a) Different weight ratios of FGO was studied and tested. (b) Simultaneous measurement of the thermistors encapsulated with different weight ratios of FGO.

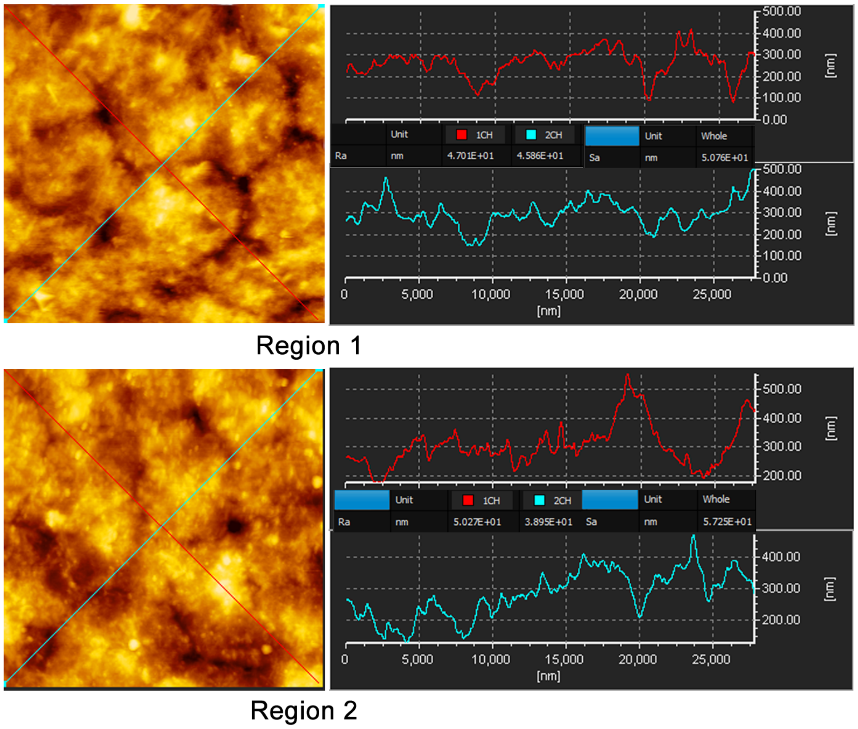

Fig. S4 AFM measurement of encapsulated layer to examine the surface morphology in two different regions (Region 1 and Region 2) having an area of 20 x 20 µm^2.

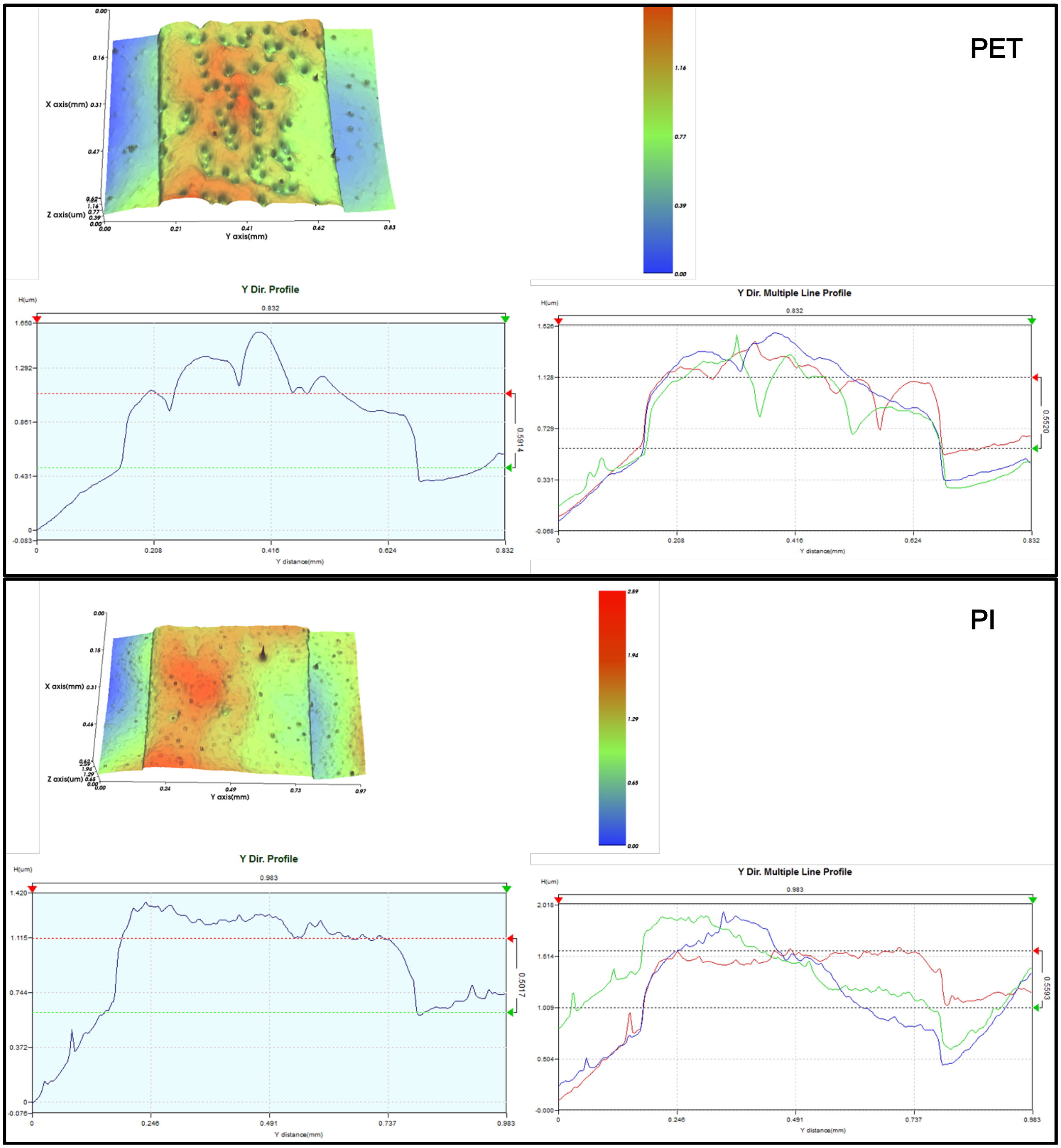

Fig. S5 Thickness of R2R printed NFC antenna patterns in PET (top) and PI (down) rolls, respectively.

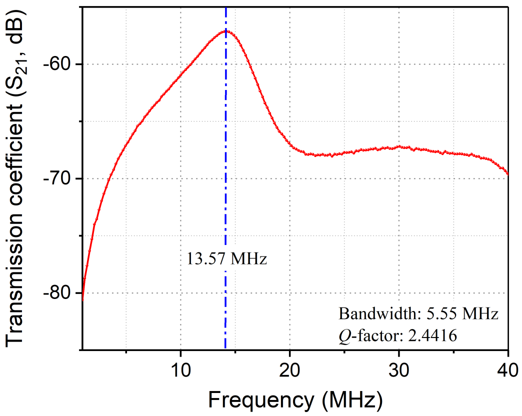

Fig. S6 Resonance characteristics Q- factor measurement of the R2R gravure printed antenna to tune at 13.56 MHz.

Table S1 Antenna dimensions (mm).

Design		Number of turns	Length (L)	Breadth (B)	Gap (g)	Width (w)
1	A, B, C, D	4	89.24	48.25	0.55	1.2
2	A, B	5	60	40	0.40	1.0

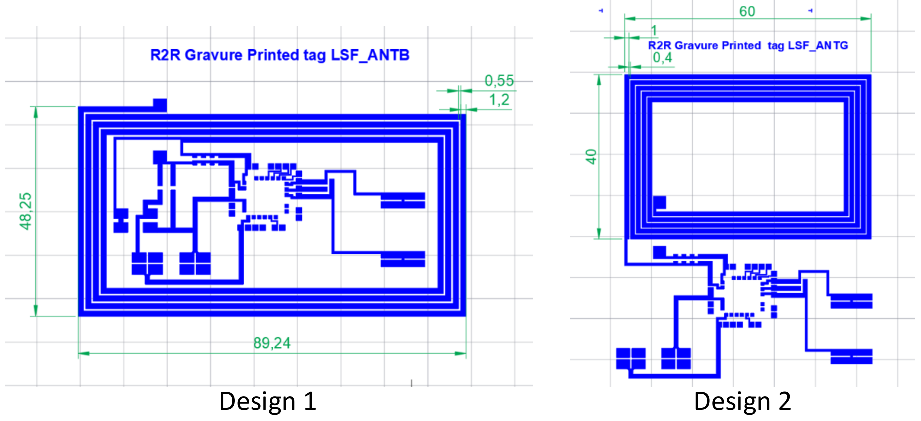

Table S2 Cost estimation of the smart label considering for mass production by R2R gravure printing.

Items	Price (in $)	Amount	Price per unit	Price per label (in $)	Remarks
Si-chip*	0.12*	Per piece	0.12	0.12	*
Resistors and capacitors	0.005	Per set	0.005	0.005	One set: 9-capacitors; 2-resistors
Printed battery	0.5	Per piece	0.5	0.5	Can be scaled down in the future.
PEDOT:PSS	1500	1000 g	1.5 per mg	0.00023	Estimated: 0.15μL per design
FGO-PVDF	100	25 mL	4 per mL	0.0072	Estimated: 1.8 μL per design
Silver ink	650	1000g	0.00076 per mg	0.0005	Estimated: 0.76 μL per design
Miscellaneous (Glue, and daughter board, and substrate)	0.05	0.05	0.05	0.05	
**Total (price per label)**				0.7	

*a Even though we used RF430FRL152H, Texas Instrument chip costing >$5.0 per unit in this work, replacing this Si-chip with low-cost alternatives (such as NT3H1201W0FHKH, NXP Semiconductors) would significantly reduce the cost which will be $0.12 per chip for mass production.
